# Supplementary material for: Effects of Complex DNA and MVs with GTF Extracted from Streptococcus mutans on the Oral Biofilm
Source: Molecules. 2019 Aug 28;24(17):3131. doi: 10.3390/molecules24173131 (PMC6749223; doi:10.3390/molecules24173131)
Supplement: Supplementary file 1 [file molecules-24-03131-s001.pdf]

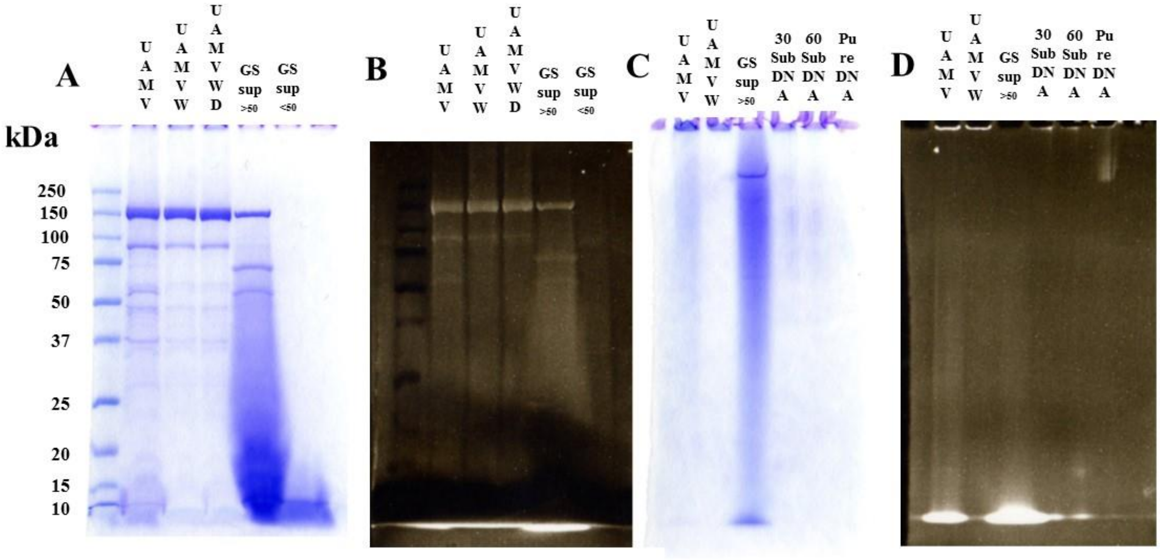

Fig. S1. Effects of DNase I on biofilm of *S. mutans* formed by MVs

MVs, MVs washed with sterile PBS (MVW) and MVW treated with DNase I (MVWD) from UA159, and culture supernatant without MVs, >50 kDa (GSsup>50) and <50 kDa (GSsup<50) from GS-5 were added to SDS-PAGE, and gels were stained with CBB (A) and ethidium bromide (B). M: Protein marker. UAMV, UAMVW, GS sup>50, subpurified DNAs prepared by cell lysis by bead destruction for of 90 seconds (90 Sub DNA) and 180 seconds (180 Sub DNA), and pure DNA from UA159 were added to native-PAGE, and gels were stained with CBB (C) and ethidium bromide (D). Representative data from more than three independent experiments are shown in the pictures.

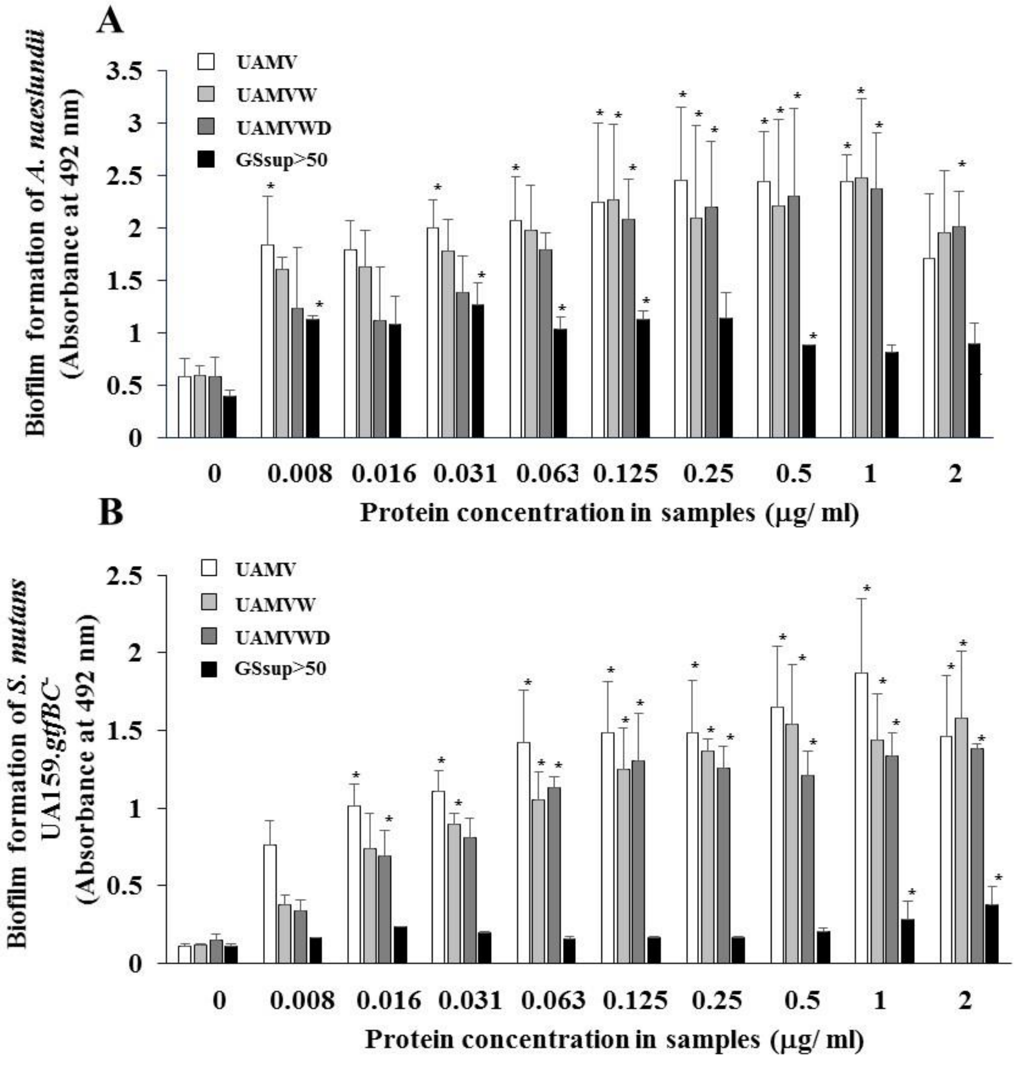

Fig. S2. Biofilm formations by MV, MVW, MVWD and GSsup>50

Effects of UAMV, UAMVW, UAMVWD from Uand GSsup>50 in Fig. 5 on biofilm formation by *S. mutans U*A159.*gtfBC*$^-$ (A) and *A. naeslundii* x600 (B) were observed at various protein concentrations. The data indicate the mean ± standard deviation (SD) of triplicate experiments. The experiments were performed three times, with similar results obtained each time. The asterisks indicate a significant difference between the two groups (*: $p < 0.05$, samples vs no sample)

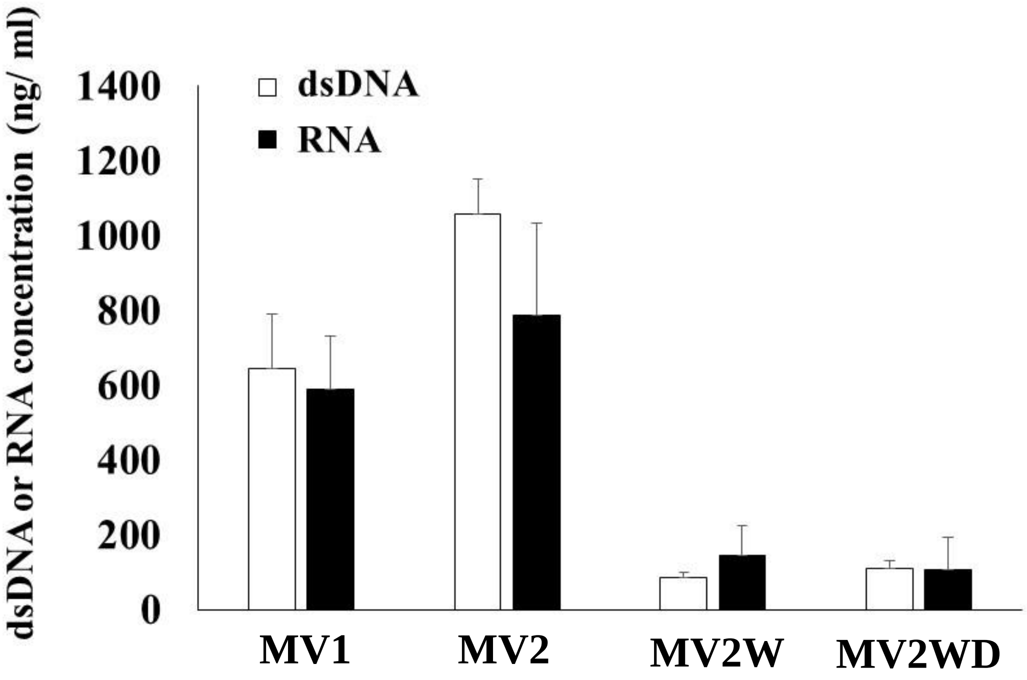

Fig. S3. Quantitative analysis of double strand DNA and RNA in MVs

Two types MVs (MV 1 and MV 2) from UA159 were extracted from culture supernatant concentrated by centrifugal filters for >10kDa and >50kDa. MV2 were washed with sterile PBS (MV2W) and MV2W was treated by DNase I (MV2WD). Double strand DNA and RNA were quantitatively measured by NanoDrop in MV samples. The data indicate the mean ± standard deviation of triplicate experiments. The experiments were performed three times, with similar results obtained in each.
